# Evaluation of a University’s Institutional Review Board Based on Campus Feedback: A Cross-Sectional Study

**DOI:** 10.7759/cureus.5829

**Published:** 2019-10-03

**Authors:** Mohammad Hasan Rajab, Muhammad Z Alkawi, Abdalla M Gazal, Faizah A Alshehri, Hassan S Shaibah, Lisa Doraine Holmes

**Affiliations:** 1 Biostatistics, Epidemiology, and Public Health, College of Medicine, Alfaisal Univerity, Riyadh, SAU; 2 Neurology, King Faisal Specialist Hospital and Research Center, King Abdul Aziz City for Science and Technology, Riyadh, SAU; 3 Public Health, Alfaisal University, Riyadh, SAU; 4 Biomedical Sciences, Alfaisal University, Riyadh, SAU; 5 Medical Education, Alfaisal University, Riyadh, SAU; 6 Public Affairs, University of Texas at Dallas, Allen, USA

**Keywords:** institutional review board (irb), research ethics, academic institution, cross-sectional study, alfaisal university, kingdom of saudi arabia, non-academic institution, campus feedback

## Abstract

Introduction

Maintaining research ethics within a university and monitoring the campus Institutional Review Board (IRB) are essential responsibilities not to be taken lightly. IRBs occasionally need to be reviewed to see that they, as well as researchers, are adhering to rules and regulations on ethics through their submission and review procedures. Since there are no established measures for assessing IRB quality, it is unclear how to determine whether IRBs are achieving their intended aims. This study used the feedback and input of campus members at a newly-established, private, non-profit university within the Kingdom of Saudi Arabia (KSA) to evaluate their campus IRB.

Methods

Following the university’s IRB approval, and in close collaboration with the Saudi National Committee of Bioethics (NCBE), this cross-sectional study was conducted from February through May of 2019. Self-administered surveys were sent out via university emails to faculty and students at Alfaisal University in Riyadh of Saudi Arabia. The questions in the surveys included inquiries on participants’ demographics, their familiarity with campus IRB research ethics, their satisfaction with IRB procedures, the challenges encountered during the IRB submission and review process, the effectiveness of a recent IRB-coordinated research ethics campaign, and any suggestions for IRB improvement. Surveys were sent to faculty members and students at five colleges on campus.

Results

Of the campus members who were sent surveys, 8% responded (175). Of those who responded, 29.7% had submitted at least one research proposal for IRB review during the past three years (2016-2019), and more than half of this group were satisfied with the IRB submission and review procedures. For those who had submitted at least one research proposal, respondents reported the more usual challenges that researchers tend to encounter, such as time-consuming and tedious IRB review processes and ambiguous IRB guidelines and regulations. The less typical IRB challenges that were reported, and that are unique to academia, include the IRB tendency to deny undergraduate student requests to serve as principal investigators of their research projects. Concerning IRB efforts to educate and train campus members on research ethics, only 26.3% of the participants were aware of the recently performed research ethics campaign, and 7.6% of the participants attended the end-of-campaign workshop. Of those who attended the workshop, 76.9% reported that the campaign and workshop effectively met their expectations.

Conclusions

This study revealed several issues encountered by university faculty and students seeking campus IRB approval for their research projects. The main academia-specific challenge was over whether undergraduate students could serve as PIs for research projects, and a universal one was that they find the IRB process to be very time-consuming and tedious, which is a situation that has already been relayed in several other articles on IRB issues. About two-thirds of respondents reported a lack of familiarity with the topic of research ethics. This challenge makes it clear that information on research ethics is not effectively reaching enough campus members in the busy environment of academia.

## Introduction

As the 20th century drew to a close, the Saudi Government, in collaboration with several private Saudi research institutions, began to focus more intently on cultivating the nation’s ability to conduct biomedical research [1]. Their efforts were met with success, with a significant increase in the number of biomedical research projects being conducted across the country [1]. To guard against the potential for misconduct and living creature abuses, the Saudi National Committee of Bioethics (NCBE) was instituted in 2001 by a Royal Decree [1]. The NCBE, consisting of designated national bioethics experts and seasoned researchers, was given a mandate of setting the ethics standards for research on living creatures and monitoring their implementation throughout the Kingdom [1].

Following the establishment of the NCBE, all Saudi institutions researching living organisms were required to comply with the policies and regulations issued by the NCBE. One of these regulations mandates that each Saudi institution engaged in the research of living creatures establish an Institutional Review Board (IRB).

An IRB is a committee sanctioned by law or regulations to approve and monitor biomedical research projects after determining whether the research project follows the ethical principles and regulations for the protection of human subjects [2]. An IRB emphasizes the upholding of basic ethical research standards to prevent the reoccurrence of past research misconduct practices, including abuses of human research subjects [3-4]. IRBs are also referred to as Research Ethics Committees (RECs) or Local Ethics Committees (LECs) [1,5].

Each IRB committee consists of at least five members of diverse scientific specializations who are interested in research ethics, with at least one member who is not affiliated with the institution to represent the interests of the community [1]. IRB committees must meet periodically to review and ascertain the acceptability of research protocol submissions using a set of written rules and procedures. The procedures themselves consist of several steps that may take a few days to a few weeks to conclude, depending on the completeness of the submitted documents, the type of IRB review (i.e. exempted, expedited, or full), the study design, the willingness of the principal investigator (PI) to fully engage in the review process, and the time of the submission (e.g., close to an exam time or national holiday).

During the IRB review process, researchers may face certain issues or challenges. For example, the IRB procedures may differ among other institutions, and some researchers may come from different national and international academic IRB systems, thus bearing different experiences with IRBs’ policies and procedures [6-8]. Such IRB differences may lead to confusion and frustration among these researchers. Another challenge some academic researchers have experienced was that some review processes were extended or delayed when significant burdens were placed on IRB administrative time and resources, especially for specialized research projects [2,5]. In particular, clinical trials must go through a more in-depth IRB review process than other study designs before the commencement of any research activity [9]. Consequently, some researchers are questioning whether the IRB, in its efforts over the years to protect study subjects, has exceeded its mandated authorities [10].

To help Alfaisal University faculty and students better understand the purpose of an IRB office and to help familiarize them with its procedures, the university’s IRB staff conducted a university-wide IRB awareness campaign in early 2018. The campaign’s central theme was that all research involving living creatures conducted on campus must be reviewed, approved, and monitored thereafter by the office of the campus IRB. Campaign key features included placement of educational posters throughout the institution and use of the school email system to inform university campus members of the campaign and IRB mandates, procedures, and activities. On the last day of the campaign, campus members were invited to a workshop, held in association with the NCBE, which provided an overview of the policies and regulations of the NCBE and ended with an open discussion session.

Taking into account that IRBs may err now and then, periodic evaluations are warranted [11]. IRBs occasionally need to be reviewed to see that they, as well as researchers, are adhering to rules and regulations on ethics through their submission and review procedures. Since there are no established measures for assessing IRB quality, it is unclear how to determine whether IRBs achieve their intended aim [12]. Evaluations of IRBs may come from sources such as government regulatory agencies or committees, school administrators, and academic researchers themselves. In this case, our study uses the feedback and input of faculty and students at a newly-established, private, non-profit university within the Kingdom of Saudi Arabia (KSA) to evaluate the campus IRB.

Unfortunately, efforts to empirically identify and document challenges encountered by academic researchers during compliance with the IRB submission and review procedures in the KSA have been marginal at best [2]. The IRB office at Alfaisal University in Riyadh elected to conduct a study that would evaluate, through the direct experiences of its campus members, their performance based on campus feedback. More specifically, the study aimed to examine the campus IRB’s procedures by identifying issues and challenges encountered by researchers in obtaining IRB approvals from 2016 through 2019. This study also endeavored to determine whether a research ethics campaign recently conducted on campus by the IRB staff met the expectations of the faculty and students of the university. This study would also highlight the university's rapid progress in developing research ethics capacity and point to some important areas for improvement.

## Materials and methods

Following the university’s IRB approval, and in close collaboration with the NCBE, we conducted a cross-sectional study from February through May of 2019. Study investigators developed an anonymous, self-administered online survey using Google Forms.®

The survey was designed for faculty and students and contained an introductory paragraph that informed participants of the study objectives, the anonymity of their responses, and of the freedom to decline a response or to withdraw completely. Besides, the survey contained a combination of closed- and open-ended questions. The closed-ended questions included inquiries into participants’ demographics, their familiarity with campus IRB research ethics, their satisfaction with IRB procedures, the challenges encountered during the IRB submission and review process, and the effectiveness of a recent IRB-coordinated research ethics campaign. Open-ended questions included solicitations for IRB improvement recommendations.

As a part of the survey validation process, 10 university faculty were asked to pilot-test the survey draft. The pilot-test revealed, among other things, that it takes approximately 10 minutes to complete the survey.

To start the survey process, an introductory email was sent to members of the target population, which consisted of all faculty and students of Alfaisal University, informing them of the study objectives and soliciting their participation. Then the survey’s web-link was sent to the target population using the institutional email system. Subsequently, two follow-up reminders were emailed to the same group. The survey targeted all five colleges of the university, including the College of Medicine (COM), the College of Engineering (COE), the College of Business (COB), the College of Science and General Studies (COSGS), and the newly established College of Pharmacy (COP). Exclusion criteria included IRB members and staff, students who have already graduated, and faculty members who had participated in the pilot-test of the study survey.

Baseline and outcome characteristics were summarized using descriptive statistics as appropriate. Data management and analysis were performed using the Statistical Package for the Social Sciences, version 21 for Windows.

## Results

The survey outreach effort received a response rate of 8% (175). Of these respondents, 55.4% were female, 58.9% were undergraduate students, 22.9 % were faculty, 56.1% were from COM, and 64.6% of respondents reported being familiar with the general principles of research ethics. The baseline characteristics of survey respondents are summarized in Table 1. 

**Table 1:** Baseline characteristics of survey respondents

	%*	N^**^
Gender		175
Male	44.6	
Female	55.4	
College		173
Medicine	56.1	
Engineering	16.8	
Business	13.3	
Science	10.4	
Pharmacy	3.5	
Degree		175
High school certificate	41.1	
MD, MBBS	20.0	
PhD	17.7	
BSC	10.9	
Masters	8.6	
Other	1.7	
Position		175
Undergraduate student	58.9	
Faculty	22.9	
Other	18.3	
Familiarity with research ethics		175
Very familiar or familiar	64.6	
Not familiar	30.3	
No opinion	5.1	
^*^Due to rounding error, percentages may not equal 100% ^**^Numbers vary owing to differing individual survey question responses		

The study respondents were asked to evaluate the effectiveness of the university-wide research ethics awareness campaign that was implemented before the administration of the survey. Of all respondents, 26.3% reported being aware of the ethics awareness campaign, and only 7.6% attended the end-of-campaign workshop. Of those who attended the workshop, 76.9% reported that the campaign and workshop effectively met their expectations. Evaluation of the effectiveness of Alfaisal’s research ethics campaign is presented in Table 2.

**Table 2:** Evaluating the effectiveness of a university-based research ethics campaign

	%	N
Aware of the ethics campaign		175
Yes	26.3	46
No	73.7	129
Attended the end-of-campaign workshop		175
Yes	7.6	13
No	92.4	162
Campaign and workshop were effective		13
Yes	76.9	10
No	23.1	3

Merely 29.7% of the respondents had submitted at least one research proposal for IRB review during the last three years, and only those respondents were allowed to finish the survey. Out of those who completed the survey, 55.7% reported satisfaction with the IRB submission procedures, and 50% reported being satisfied with the IRB review procedures. The main reported reasons for dissatisfaction with the IRB review procedures included the time it took to complete the review process and to respond to the unclear or unexplained IRB review critiques. Experience with the campus IRB submission and review procedures is presented in Table 3. 

**Table 3:** Respondents’ experience with the campus Institutional Review Board submission and review procedures

	N*	%
Submitted at least one proposal to the IRB		175
Yes	29.7	
No	70.3	
Satisfaction with the IRB submission procedures		52
Very satisfied or satisfied	55.7	
Neutral	32.7	
Dissatisfied or very dissatisfied	11.6	
Satisfaction with the IRB review procedures		52
Very satisfied or satisfied	50.0	
Neutral	26.9	
Dissatisfied or very dissatisfied	23.1	
Reasons for dissatisfaction with IRB review procedures^**^		19
Review time	78.9	
Unexplained criticism	73.7	
Suggestions difficult to implement	26.3	
Other	15.8	
^*^Numbers vary owing to differing individual survey question responses ^**^Respondents checked all that apply		

Respondents who submitted proposals to the IRB during the period 2016-2019 reported several issues or challenges that were experienced during compliance with IRB’s procedures. These challenges included: (1) technical difficulties with the online submission procedures; (2) time-consuming and tedious IRB review procedures; (3) occasional unavailability of qualified experts to review specialized topics; (4) decisions by IRB against permitting undergraduate students to serve as PIs for their studies; and (5) lack of familiarity with the principles of research ethics (1/3 of respondents).

Further, respondents suggested several ways to improve IRB procedures and functions including (1) higher efficiency of the IRB submission and review process; (2) a reduction in IRB submission review time by focusing the review process solely on the principles of ethics of research. That is, the evaluation of the scientific merits aspect of submitted proposals would be assigned to college scientific review (or research) committees; (3) the training of the undergraduate students to serve as PIs under the supervision of qualified faculty; and (4) the availability of periodical and effectively-announced IRB-related education courses and awareness-raising campaigns. Reported concerns and challenges that were experienced during compliance with IRB’s procedures and recommended actions are summarized in Table 4.

**Table 4:** Reported concerns and challenges that were experienced during compliance with Institutional Review Board’s procedures and recommended actions

Concerns/challenges	Recommended actions
Time-consuming and tedious IRB review process	(1) Advise researchers at the study design stage, (2) Improve communication efforts with PIs, (3) Focus more on the principles of ethics and less on scientific merits of a study, (4) Increase IRB membership, (5) Refer evaluation of studies’ scientific merit to college research committees
Unavailability of sufficient number of qualified experts to review specialized topics	Solicit the support of experts from the National Committee of Bioethics (NCBE)
Undergraduate students precluded by IRB from serving as principal investigators	Allow undergraduate students to serve as PIs for their studies if supervised by qualified faculty and received training in basic research and research ethics
Unfamiliarity by some campus members with the principles of research ethics	Conduct periodic research ethics awareness activities, training, and educational programs

## Discussion

Low response rate 

Several reasons may have contributed to such a low response rate. First, the study was conducted during a busy mid-term season. Second, most respondents are from COM, which is the college with the highest number of faculty members and students. Students of COM are generally swamped with academic and clinical coursework. However, the 3.4% response rate of the COP group was most likely low because enrollment at the three-year-old college is still growing. Last but not least, social media and smartphones, some of the most effective methods of communicating with the university population, were not fully utilized in this study [13]. University faculty and students receive many emails daily and may not always have the opportunity to read incoming messages, especially those unrelated to the coursework.

Academic vs. non-academic institutions

Notwithstanding the low response rate, this study does identify several challenges encountered by academic researchers during the IRB process, and it also makes a distinction between academic and non-academic settings for such challenges. It is essential to learn the extent to which the information acquired in this academic-oriented study is generalizable to non-academic institutions such as pharmaceutical companies, biomedical research centers, and hospitals.

For example, study results show that about two-thirds of study participants have not submitted a research proposal to the IRB during the past three years. If the study population is to be representative of the entire campus, then two-thirds of the campus population most likely possess limited knowledge of IRB regulations and procedures compared with the experience of most researchers in non-academic settings. A closer look at the types of studies being done at academic institutions also helps us to understand the difference in the levels of research experience. IRB records at Alfaisal show that during the period between 2016 and 2019, 80% of the research proposals submitted for IRB reviews were cross-sectional studies, which are studies that take less time for IRBs to review and process than the time required for clinical trials. In non-academic institutions, clinical trials and other similar study designs of greater complexity have far more commonly endeavored. Findings by Brew and Lucas indicate that research studies conducted at academic institutions and those conducted at non-academic institutions do differ in types of study designs utilized [14].

For other challenges unique to academia, both student and faculty respondents pointed to the tendency of IRB to decide against allowing undergraduate students to serve as PIs for their studies. Understandably, this issue would surface during the study. Three related problems have been revealed through informal communication with campus students concerning the IRB delegation of study PIs: (1) By not serving as PIs for their studies, students are unable to receive full credit for their research work, (2) Not enough faculty members are available to serve as PIs for the numerous student studies, and (3) Even when students are permitted to serve as the PI on the study, faculty members who work with the student(s) may still exercise undue influence on the construction and outcome of the student’s study. One recent study, in particular, points to several critical ambiguities and dilemmas in how IRBs view and approach questions about faculty coercion and undue influence [15]. This is a challenge that is experienced in academia worldwide.

Respondents who have interacted with the campus IRB during the past three years reported challenges similar to those already reported in biomedical literature [5]. For example, one such challenge was that researchers found the IRB review process to be time-consuming and tedious. It is worth mentioning, however, that almost half of the respondents reported satisfaction with the ease, clarity, promptness, and appropriateness of the campus IRB submission and the review procedures.

IRB actions

In response to the information and recommendations acquired from the study, IRB plans at Alfaisal are now underway to improve IRB functions and awareness/training campaigns. For example, a time-efficient submission and review model will be tested based on researchers’ feedback. Another idea under consideration is to involve IRB members or research ethics experts at the study design stage so that investigators are being assisted in developing ethical and regulatory-compliant research proposals [9].

Usually, the Alfaisal IRB examines the scientific merit of the submitted proposals to ensure that research is of the utmost quality, and it does so by consulting with scientific experts [7]. However, many of the respondents did recommend that such a practice be discontinued because it tends to lengthen the time required for the review process. They argued that the main function of an IRB is really to protect study participants through research ethics. In response to this point, the campus IRB is considering a plan to defer the review of scientific merit within research studies to college research committees. Unfortunately, this extra step may still require additional time which may further delay the IRB review process.

IRB awareness

The respondents also recommended that the campus IRB periodically coordinate some research ethics training and awareness activities. As it is, Alfaisal IRB staff has been unable to take full advantage of the opportunity at fall-time orientations for new academia arrivals, or at other events, to provide sufficient information about IRBs.

Only about a quarter of respondents were aware of the ethics awareness campaign, and even fewer attended the end-of-the-campaign workshop. However, most of those who attended the workshop were satisfied with these activities. The opportunity for these participants to have a question/answer session with biomedical research ethics experts from the NCBE and the university may have contributed towards their satisfaction.

IRB training

One of the responsibilities of an IRB office is to train researchers in research ethics [16]. A study from John Hopkins University on the emerging experiences of researchers at Botswana University demonstrates the importance of this training. As is the case at Alfaisal University, their case study was an initial effort to document researchers’ ethics outreach and education in a context where both research and research oversight are relatively new. Findings revealed that researchers indeed recognized the critical need for research ethics training and that ethics review processes can help researchers better understand and value research ethics [17].

In response to our study participants’ suggestion, Alfaisal’s campus IRB plans to commit to periodical university-wide research ethics training and awareness-raising activities. Moreover, plans are in motion for a representative of the IRB to attend the next school orientation held for new faculty and students where he or she will convey the essential IRB information followed by a “question-and-answer” session. The IRB also intends to engage the university faculty and students in a series of lunch-hour auditorium seminars complete with discussion sessions focusing on the principles of research ethics and the role of the university IRB. Recommendations for campus IRB improvements similar to those garnered in our study, including a more efficient IRB process, were also highlighted by Moon and Khin-Maung-Gyi [18].

Study limitations

The low response rate of our IRB study may be the result of being a single-site study, and the result of the decision not to utilize social media in the awareness campaign. Another study limitation is recall bias. Consequently, the study may have limited generalizability [19]. Despite these limitations, the study does provide us with relevant knowledge of the challenges facing today’s academic researchers and their campus IRBs that may be evident in other academic institutions.

Future plans

Identification of the challenges encountered by researchers seeking IRB approvals for their projects is a necessary step in testing and developing specific solutions to improve IRB procedures [19]. However, while evaluating the review process of an IRB is challenging, evaluating procedures of a newly established IRB can be especially challenging [20]. Thus, plans include further studies using on-campus IRB evaluations from participants at several academic institutions. Recruitment should be administered using a wide selection of available means of communication, including social media, to enhance the generalizability of research results [21].

## Conclusions

Several persistent challenges experienced by Alfaisal University faculty members and students while working with the campus IRB had become evident by the end of the study. Some of these issues are unique to academia. The main academia-specific challenge was over whether undergraduate students could serve as PIs for research projects, and a universal one was that they find the IRB process to be very time-consuming and tedious, which is a situation that has already been relayed in several other articles on IRB issues. About two-thirds of respondents reported a lack of familiarity with the topic of research ethics. This challenge makes it clear that information on research ethics is not effectively reaching enough campus members in the busy environment of academia.

Therefore, institutions are advised to maintain periodical evaluations of IRB procedures to (1) identify the challenges experienced by academic staff, and (2) recommend ways of resolving those issues. This study is expected to stimulate greater interest among members of the international research community, particularly those on campuses, in the development of IRB measurements of compliance and quality control.
